# Potential biomarker of brain response to opioid antagonism in adolescents with eating disorders: a pilot study

**DOI:** 10.3389/fpsyt.2023.1161032

**Published:** 2023-07-10

**Authors:** Stephani L. Stancil, Hung-Wen Yeh, Morgan G. Brucks, Amanda S. Bruce, Michaela Voss, Susan Abdel-Rahman, William M. Brooks, Laura E. Martin

**Affiliations:** ^1^Divisions of Adolescent Medicine and Clinical Pharmacology, Toxicology and Therapeutic Innovation, Children’s Mercy Kansas City, Kansas City, MO, United States; ^2^Department of Pediatrics, University of Missouri-Kansas City School of Medicine, Kansas City, MO, United States; ^3^Department of Pediatrics, University of Kansas Medical Center School of Medicine, Kansas City, KS, United States; ^4^Division of Health Services and Outcomes Research, Children’s Mercy Research Institute, Kansas City, MO, United States; ^5^Department of Population Health, University of Kansas Medical Center, Kansas City, KS, United States; ^6^Center for Children’s Healthy Lifestyles and Nutrition, Kansas City, MO, United States; ^7^Department of Neurology, University of Kansas Medical Center, Kansas City, KS, United States; ^8^Hoglund Biomedical Imaging Center, University of Kansas Medical Center, Kansas City, KS, United States

**Keywords:** opioid antagonism, eating disorders, naltrexone, adolescents, pharmacodynamic biomarker, fMRI

## Abstract

**Background:**

Eating Disorders (ED) affect up to 5% of youth and are associated with reward system alterations and compulsive behaviors. Naltrexone, an opioid antagonist, is used to treat ED behaviors such as binge eating and/or purging. The presumed mechanism of action is blockade of reward activation; however, not all patients respond, and the optimal dose is unknown. Developing a tool to detect objective drug response in the brain will facilitate drug development and therapeutic optimization. This pilot study evaluated neuroimaging as a pharmacodynamic biomarker of opioid antagonism in adolescents with ED.

**Methods:**

Youth aged 13–21 with binge/purge ED completed functional magnetic resonance imaging (fMRI) pre- and post-oral naltrexone. fMRI detected blood oxygenation-level dependent (BOLD) signal at rest and during two reward probes (monetary incentive delay, MID, and passive food view, PFV) in predefined regions of interest associated with reward and inhibitory control. Effect sizes for Δ%BOLD (post-naltrexone vs. baseline) were estimated using linear mixed effects modeling.

**Results:**

In 12 youth (16–21 years, 92% female), BOLD signal changes were detected following naltrexone in the nucleus accumbens during PFV (Δ%BOLD −0.08 ± 0.03; Cohen’s *d −*1.06, *p* = 0.048) and anterior cingulate cortex during MID (Δ%BOLD 0.06 ± 0.03; Cohen’s *d* 1.25, *p* = 0.086).

**Conclusion:**

fMRI detected acute reward pathway modulation in this small sample of adolescents with binge/purge ED. If validated in future, larger trials, task-based Δ%BOLD detected by fMRI may serve as a pharmacodynamic biomarker of opioid antagonism to facilitate the development of novel therapeutics targeting the reward pathway, enable quantitative pharmacology trials, and inform drug dosing.

**Clinical trial registration:**

https://clinicaltrials.gov/ct2/show/NCT04935931, NCT#04935931.

## Introduction

Eating disorders affect up to 5% of youth regardless of gender, race, and ethnicity, have their onset in adolescence and are associated with substantial morbidity and mortality (1–4). Many eating disorders are characterized by impulsive and compulsive behaviors such as binge eating and/or purging seen with Anorexia Nervosa–Binge/Purge (AN-BP), Bulimia Nervosa (BN), Binge Eating Disorder (BED) and Other Specific Feeding and Eating Disorders (OSFED). These conditions carry increased risk of cardiac compromise, metabolic dysfunction, substance use disorder, and suicidality (3, 4). Although two medications are approved for use in adults (fluoxetine in BN, lisdexamfetamine in BED), there are no approved medications for the treatment of eating disorders in adolescence. Moreover, data supporting safe and effective use of these or other medications in adolescents are lacking.

Reward pathway alterations have been detected in patients with binge/purge related eating disorders. Neuroimaging evidence suggests alterations in reward-based learning and reinforcement (5) as well as reward sensitivity and regulatory control (5). Most published studies point to reduced reward sensitivity, consistent with a hypodopaminergic state and suggest that individuals with binge/purge eating disorders are motivated toward dopamine-seeking behaviors to reach reward homeostasis (6–8). A small study of adults suggests that reduced μ opioid receptor (MOR) binding in the insula, the main region associated with taste and also thought to contribute to anticipation and reward of eating, is associated with BN compared with healthy controls (9). Other fMRI studies of adults with BN and BED point to increased reward sensitivity to food images and delivery relative to healthy controls that may be related to hunger (increased activation in the medial orbitofrontal cortex (mOFC), anterior cingulate cortex (ACC) and insula) (10, 11) and altered regulatory control (decreased activation in frontal striatal circuits, lower ACC activation) (12, 13). Further, individuals with binge/purge related eating disorders demonstrate high trait impulsivity, a feature also present in individuals with substance use disorders (14). Taken together, these findings suggest that patients with binge/purge eating disorders do have altered reward system function, but the specific mechanisms have yet to be fully elucidated.

Targeting reward alterations is a promising avenue for treatment. Naltrexone is a non-selective opioid antagonist, approved for treating alcohol and opioid use disorder in adults, and is used to target binge eating and/or purging in patients with eating disorders. Small studies suggest the potential for effectiveness in eating disorders (15). The proposed mechanism of action is blockade of MOR-mediated euphoria that would otherwise result from the binge eating and/or purging. Interestingly, naltrexone 50 mg administered orally has demonstrated near complete blockade of MOR in humans, yet dosages nearly 8-fold higher have been required to reduce binge eating and purging. Naltrexone also antagonizes κ (KOR) and δ opioid receptors (DOR), but with less affinity relative to MOR (16, 17). This opens the possibility that mixed antagonism across the opioid reward system may be needed for drug efficacy in the eating disorder population. Elucidating and detecting the mechanism associated with the target behaviors of binge eating and purging is needed to inform optimal dose selection and identify new avenues of treatment.

Objective and sensitive indicators are the ideal tool to detect response to therapeutic interventions. Unfortunately, the field of neuropsychopharmacology is limited by lack of objective endpoints or response biomarkers. Existing endpoints rely on self-report and recognition of a collection of symptoms, which may be subject to various sources of bias (e.g., recall, social desirability, confirmation). A pharmacodynamic (PD) biomarker is a response biomarker that detects biological activity of the medication through detection of target engagement (e.g., molecular lock-and-key), pathway modulation, or disease-related change (e.g., hemoglobin A1c). PD biomarkers can improve the therapeutic landscape by enabling a quantitative pharmacology approach to drug development, drug repurposing, and dose selection (18). Though several have been explored, there are no validated PD biomarkers for use in neuropsychopharmacology (19). Better tools are needed to detect objective response in the brain and support drug development and optimization.

Functional magnetic resonance imaging (fMRI) offers some promising characteristics for a PD biomarker. fMRI is a non-invasive, non-radioactive tool that allows for the detection of blood-oxygenation level dependent (BOLD) changes in the brain which serve as a proxy for neuronal activation. Additionally, fMRI allows for localization of brain activity in specific regions of interest and identification of coordinated brain activity within networks. Neurobehavioral probes can be used to elicit activation of a particular brain pathway, which allows for the study of pathway regulation and manipulation. fMRI has detected changes in reward system activation following naltrexone in adults with obesity and alcohol use disorder (20–22); however, no such data exist in adolescents. Adolescence is a dynamic period of brain development represented by an imbalance between the robust function of reward circuitry (e.g., nucleus accumbens) and the relatively underdeveloped cortical regions involved with executive function and inhibitory control (e.g., prefrontal cortex (PFC)). This “mismatch” is thought to lead to overvaluing short-term, high reward scenarios and undervaluing potential negative consequences. fMRI has the potential to detect opioid reward pathway modulation and may serve as a useful, non-invasive tool to study drug response in the adolescent brain and inform dose selection. This neurodevelopmental difference between adolescents and adults underscores the importance of studying this population. The purpose of this pilot study was to evaluate fMRI as a pharmacodynamic biomarker of acute opioid antagonism in adolescents with eating disorders. We aimed to determine the extent of reward pathway modulation and hypothesized that acute opioid antagonism would increase activation in the ACC.

## Materials and methods

### Study design

This was a single arm, pre/post study in youth aged 13–21 years with eating disorders characterized by binge eating and/or purging according to the Diagnostic and Statistical Manual of Mental Disorders–5th edition (DSM-V) (e.g., Anorexia Nervosa-Binge/Purge, Bulimia Nervosa, Binge Eating Disorder) (23), as diagnosed by their treating clinician (e.g., adolescent medicine clinician or licensed psychologist). Adolescents were eligible for enrollment if they were (1) not currently taking naltrexone (within ≥4 weeks), (2) on a stable regimen if on other medications (no dose/drug changes ≥4 weeks), (3) had no opioid exposure in the past 7 days, (4) no prior hypersensitivity reaction to naltrexone, and (5) not pregnant. Standard MR safety screening was completed for all participants and participants who failed safety screening were excluded. The study was approved by the Institutional Review Boards at Children’s Mercy Kansas City and the University of Kansas Medical Center.

### Sample size

To demonstrate feasibility of design and recruitment, we enrolled 13 participants. There is a paucity of data regarding within-subject reward system modulation (e.g., Δ%BOLD pre/post opioid antagonism). Although one study (24) reported within individual change in *n* = 3, they did not describe the regions of interest (ROI) from which the change was derived; thus, these pilot data are critical to informing validation study design.

### Assessments

Anthropometrics (including blind weight), vital signs, and medical history were recorded. The validated Eating Pathology Symptom Inventory (EPSI) was used as a proxy for eating disorder disease status at time of visit (25, 26). Individual scores were transformed to age- and sex-specific percentiles based on published eating disorder normative data (26). Before each scan, hunger was assessed using a 4-point Likert scale previously used in fMRI studies of adolescents with eating disorders (27). Safety was monitored through self-report of physical symptoms that may occur after naltrexone administration, assessed via structured questionnaire e.g., headaches, dizziness/faintness, stomach discomfort, nausea, on a 0 (no symptoms) to 4 (very severe symptoms) scale (28). If physical symptoms were experienced (score ≥ 1), the subjective experience was assessed, “overall, how distressing do you find these symptoms?” on a 0 (not distressing at all) to 4 (very distressing) scale (29).

### Standardized meals

Participants ate a standardized meal designed by an eating disorder nutritionist 1 h before each fMRI. The standardized meal contained 525 calories (28% fat, 55% carbohydrate, 18% protein).

### Study medication and MRI image acquisition

Participants completed two fMRI scans in the fed state (see Standardized Meals section) during the study day. The first scan (‘pre’) was completed before taking naltrexone. The second scan (‘post’) was completed 2 h after taking a single oral dose of naltrexone 50 mg (dispensed by the Investigational Drug Pharmacy and administered by licensed nursing staff). Naltrexone 50 mg was chosen because it is the commercially available oral dose and has been used in the treatment of adolescent binge/purge eating disorders (30). Scanning 2 h post-naltrexone was chosen because it corresponds to the expected time to maximum plasma concentration of naltrexone based on our prior work in the eating disorder population (31). Scans lasted approximately 45 min and included two runs each of two reward activation paradigms (monetary incentive delay–MID and passive food view–PFV) within 12 min of each other (32–36). Because naltrexone opioid receptor binding is persistent up to 24 h, the same order was used for each participant rather than counterbalancing. Imaging was performed on a 3 Tesla Siemens Skyra scanner using a 32-channel head coil. All participants were positioned with anterior commissure–posterior commissure plane between 4° and 20° to the scanner coordinate space to standardize participant placement and optimize signal in ventromedial prefrontal cortex (33, 37, 38). Following automated scout image acquisition and shimming procedures to optimize field homogeneity, participants completed two runs of resting state fMRI and reward-related task fMRI. Scanning procedures were adapted from the ABCD protocol (39) for the Skyra scanner: Resting state FC (TR/TE = 800/30 ms, flip angle = 52, FOV 720; slice acceleration factor 6; slice thickness = 2.4 mm, in-plane resolution = 2.84 mm, for 383 volumes), MID (TR/TE = 800/30 ms, flip angle = 52, FOV 720; slice acceleration factor 6; slice thickness = 2.4 mm, in-plane resolution = 2.84 mm, for 411 volumes), PFV: (TR/TE = 3000/25 ms, flip angle = 90, FOV 640; GRAPPA acceleration mode; slice thickness = 3 mm, in-plane resolution = 2.9 mm, for 130 volumes) (40). Field maps were acquired to correct for inhomogeneity in the BOLD data. A T1-weighted structural scan was used for spatial normalization and co-registration with fMRI data (3D MPRAGE, TR/TE 2300/2.95 ms, flip angle 9°, FOV = 256, matrix 240 × 256, slice thickness = 1.2 mm).

### Functional magnetic resonance imaging tasks

The two reward paradigms (MID and PFV) provide distinct insight into reward modulation by primary (e.g., food) and secondary (e.g., money) reinforcers associated with reward processing in different brain regions (41). Functional tasks were presented electronically using the E-Prime software (Psychology Software Tools, Pittsburgh, PA).

#### Monetary incentive delay task

MID is a widely used paradigm in adolescents to detect reward anticipation and receipt in the nucleus accumbens (NAc) and is being used to study the developmental trajectory of reward processing in hundreds of youth in the longitudinal ABCD trial (39). Using an event-related design, each trial consisted of an incentive cue (2000 ms) identifying the trial type (Win, Lose, No Money at Stake). There was a total of 100 trials (50 per run), each 3,650–6,500 ms, dependent upon cue length. The incentive cue is followed by a fixation cross (i.e., anticipation event) for 1,500–4,000 ms followed by an action cue (150–500 ms) that participants respond to with a keypress. Feedback is then presented. If they *correctly* respond to the action cue, they win money or avoid losing money (e.g., “you win $0.50” or “you did not lose $0.50”). If they *incorrectly* respond to the action cue, they either fail to win money or lose money (e.g., “you did not win $0.50” or “you lost $0.50”). Experimental contrasts of interest include: Reward Anticipation (anticipation following *win* cue minus anticipation following *no money at stake* cue), Reward Receipt (receipt of correct response to *win* trial minus receipt of response to *no money at stake* trial), Loss Anticipation (anticipation following *loss* cue minus anticipation following *no money at stake* cue), Loss Receipt (receipt of incorrect response to *loss* trial meaning money lost minus receipt of response to *no money at stake* trial).

#### Passive food view task

PFV provides a food-specific paradigm that is relevant to binge/purge behaviors, has been evaluated in adults in response to naltrexone in adults, and is expected to activate food cue-reactivity regions (e.g., prefrontal regions) (34, 35, 40, 42–45). Using a block design, participants passively viewed four 30-s blocks of food images and two 30-s blocks of nonfood images (i.e., animals) (40). Each block consists of 10 images presented for 2.5 s with a blank interstimulus interval of 0.5 s. Images were validated by Szabo-Reed et al. and selected to control for arousal and valance and differ in terms of appetizing level (35). Animal images were rated as “not appetizing.” Following each stimulus block was a 30-s block of low-level baseline images, which were the food and non-food images blurred to make images unrecognizable. This was done by applying a fast Fourier transformation (FFT), removing the phase information, and then applying the inverse FTT in MATLab (The MathWorks, Inc., Natick, MA) (35). Experimental contrasts of interest include: All Food (food vs. low-level baseline) and Animals (non-food vs. low-level baseline).

### Functional magnetic resonance imaging subject-level analysis

Data preprocessing and statistical analyzes were performed using Analysis of Functional Neuroimages (AFNI) following their standard recommendations (46). Preprocessing scripts were generated using command afni_proc.py and included motion correction, alignment, spatial smoothing, and normalization. Anatomical data were skull stripped and normalized to standard Montreal Neurological Institute (MNI) space by non-linear warping (AFNI command @SSwarper). Task-based preprocessing steps also included slice time correction which applies interpolation of each voxel time series to align the slices to the first volume of the time series. Data with motion >0.3 mm within a volume were censored from the analysis. fMRI images were realigned to the minimum outlier in each run to correct for motion. The images were spatially smoothed to 4 mm FWHM Gaussian kernel. Statistical contrasts (MID and PFV experimental conditions of interest listed above) were conducted using multiple regression analysis with motion parameters included as nuisance regressors.

For rsFC data, segmentation of the anatomical datasets was performed in Freesurfer (47) and used to estimate average signal in the ventricles and white matter. Preprocessing steps included slice time correction in addition to aforementioned steps. Data with motion >0.2 mm within a volume were censored from the analysis. To reduce spurious variance in the analysis, nuisance variables included six motion parameters (translation and rotation around x, y, z axes), average ventricle signal, and average white matter signal. The predicted time course was constructed and subtracted from each voxel time course resulting in a residual time course for each voxel. The residual time course was then smoothed with a 4 mm FWHM Gaussian kernel, resampled to a 2 × 2 × 2 mm grid and transformed to MNI space.

### Functional magnetic resonance imaging group-level analysis

**Figure 1:**
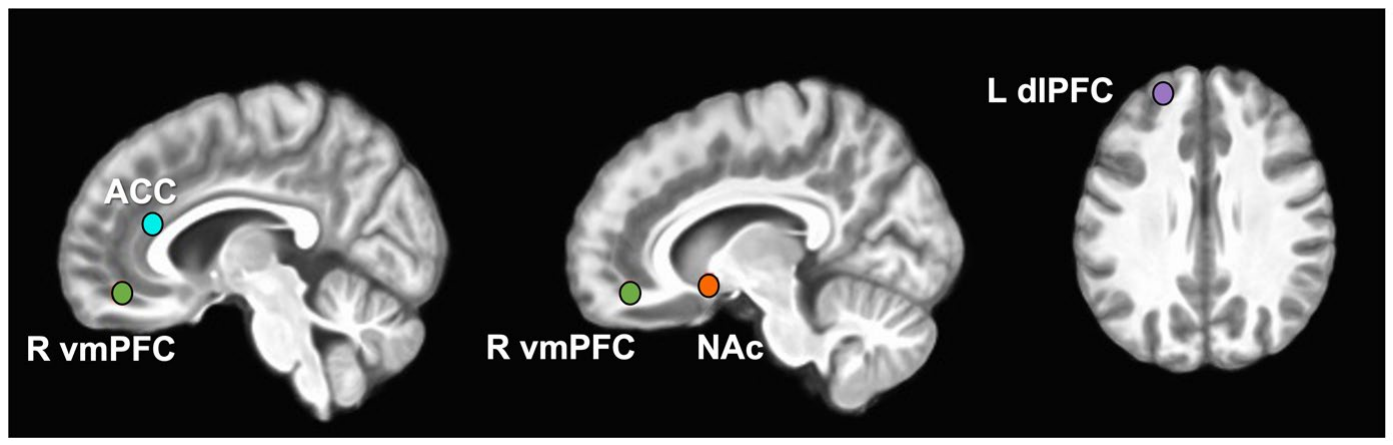
*A priori*-defined Regions of Interest (ROIs). ROIs displayed on 3D merged image from all study participant brains. MNI coordinates expressed as *x, y, z* for regions of interest: ACC (L) -4, 32, 18 (R) 4, 32, 18; dlPFC -22, 52, 30; NAc (L) -12, 8, −8 (R) 14, 10, −8; vmPFC 9, 46, −13.

Four *a priori* ROIs (ACC, dorsolateral prefrontal cortex–dlPFC, NAc, ventromedial prefrontal cortex - vmPFC) were selected from a meta-analysis of PFV and MID studies from neurosynth.org (coordinates listed in Figure 1 legend and Supplementary methods) (48). Right DLPFC and left VMPFC were not present in the results of the neurosynth meta-analysis and thus were not included as *a priori* ROIs. Spherical ROI seeds with a 5 mm radius were created around the center voxel of each ROI (See Supplementary methods for Neurosynth.org search terms). For the MID and PFV tasks, data in each ROI were scaled to percent signal change in AFNI and extracted for group analysis. For the rsFC analysis, the average time-series across the ROI was extracted for each participant and Pearson correlations were computed for the ROI pairs of interest (NAc - dlPFC and NAc - ACC) using AFNI command 1dcorrelate. This correlation coefficient was then converted to Fisher *z*-transformed values for each participant for group analysis.

### Statistical analysis

The naltrexone effects on %ΔBOLD and rsFC were assessed by linear mixed effects modeling. For each contrast of each task (listed above) in the 4 ROI (ACC, NAc, vmPFC, dlPFC) and each connectivity (NAc-dlPFC, NAc-ACC), the %ΔBOLD or the Fisher value was regressed on time (pre, post) as the fixed-effect using participant intercepts as random effects. Parameters were estimated by the restricted maximum likelihood method and degrees-of-freedom were computed by Kenward-Roger’s method for the small sample size. The fractions of uncensored fMRI data were used as the weight function in the model. For task-based %ΔBOLD, the minimal fraction of the conditions of contrasts was used as the weight (e.g., a participant had 0.55 and 0.66 uncensored data during AllFood and baseline images during the pre-scan, then 0.55 was used as the weight for the AllFood vs. baseline contrast). The fixed-effects t-statistic and the corresponding degrees-of-freedom from the linear mixed effects model were used to estimate effect sizes as Cohen’s *d,* which may inform the design of future, larger trials powered to detect an *a priori*-determined effect size. *p*-values are reported (*α* = 0.05) and correction for multiple comparison was not employed.

## Results

### Participant characteristics

Thirteen participants were enrolled and 12 completed all study procedures. One participant withdrew from the study due to claustrophobia when first introduced to the scanner and did not receive any medication or complete the scans. Data are presented for the 12 remaining participants (Table 1). No serious adverse events occurred during the study. Naltrexone was well tolerated with no adverse drug reactions.

**Table 1:** Participant characteristics (*n* = 12).

Age (y), mean ± SD (range)	17.8 ± 2.1 (16–21)
Weight (kg), mean ± SD (range)	78 ± 29 (47–148)
BMI, mean ± SD (range)	29 ± 10 (18.4–52.8)
BMI z-score, mean ± SD (range)	1.2 ± 1.2 (−1.3 to 2.8)
Gender identity, *n* (%)	
Female, cisgender	11 (92%)
Other (self-described)*	1 (8%)
Self-described race and ethnicity, *n* (%)	
Asian	1 (8%)
White	7 (58%)
More than one race	3 (25%)
Unknown/not reported	1 (8%)
Hispanic	5 (42%)
Substance use, *n* (%)	
Nicotine, daily	2 (17%)
Alcohol, regularly*	4 (33%)
Marijuana, regularly*	1 (8.3%)
Concurrent medications *n* (%)^	
SSRI/SNRI	8 (67%)
Atypical antipsychotic	2 (17%)
Hormonal contraceptives	3 (25%)
Psychiatric diagnoses, *n* (%)	
Anorexia nervosa, binge-purge	9 (75%)
Bulimia nervosa	1 (8.3%)
Binge eating disorder	2 (17%)
Major depressive disorder	10 (83%)
Anxiety disorders	11 (92%)
PTSD	3 (25%)
Bipolar	1 (8.3%)
ADHD	3 (25%)
Autism spectrum	1 (8.3%)

* Regularly defined as weekly or monthly use. No one reported daily alcohol or marijuana use.

^Medications taken by only 1 participant each: stimulant, alpha agonist and gabapentin.

ADHD, attentive deficit hyperactivity disorder; BMI, body mass index; kg, kilogram; PTSD, post-traumatic stress disorder; SD, standard deviation; SNRI, serotonin norepinephrine reuptake inhibitors; SSRI, selective serotonin reuptake inhibitors; y, years.

We compared subscale scores from the self-reported EPSI with published eating disorder norms (26). Individuals with AN-BP and BN (*n* = 10) had a mean Binge Eating or Purging score in the 65th percentile (SD 25, range 4–87) and the two individuals with BED had Binge Eating or Purging scores in the 69-87th percentiles. Individuals with AN-BP and BN (*n* = 10) had a mean Cognitive Restraint score in the 44th percentile (SD 20, range 2–71) while the two individuals with BED had scores in the 4-7th percentiles.

### Group-level reward modulation by naltrexone

**Figure 2:**
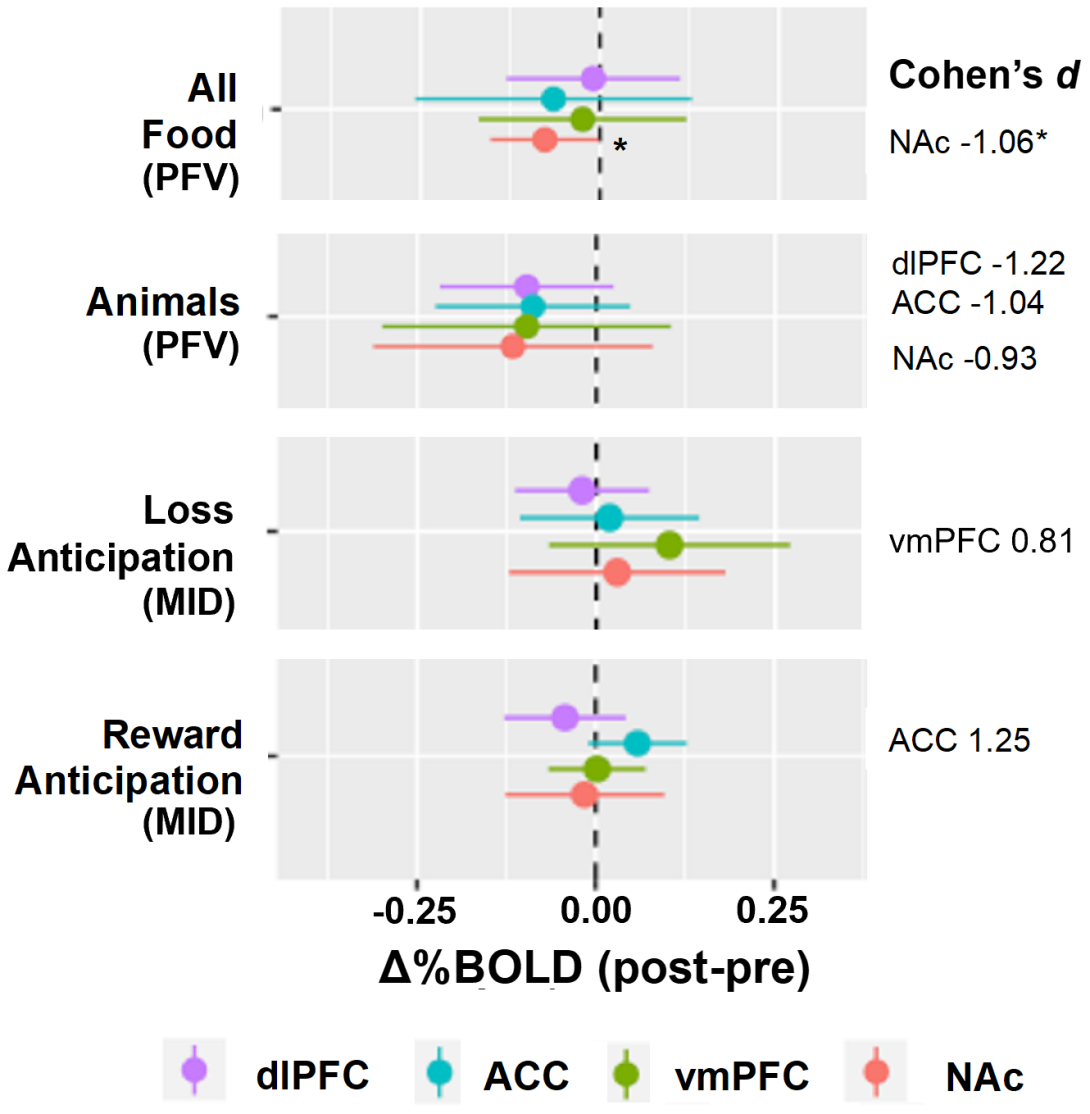
Group level reward pathway modulation by opioid antagonism. The forest plots display the linear random mixed effects model-derived mean and 95% confidence interval for %BOLD signal change following naltrexone. *A priori* regions of interest were nucleus accumbens, NAc, ventromedial prefrontal cortex, vmPFC, anterior cingulate cortex, ACC, and dorsolateral prefrontal cortex, dlPFC. Contrasts selected demonstrated Cohen’s *d* ≥ 0.8. *p < 0.05.

There were four hypothesis-driven ROIs that demonstrated a large effect of reward modulation by opioid antagonism (Cohen’s *d* ≥ 0.8; Figures 1, 2). Naltrexone reduced activation in the NAc during the PFV task, a finding that had both a large effect size and was statistically significant (Δ%BOLD -0.077 ± 0.032, *p* = 0.048). During MID, large effects were seen during anticipation of reward and loss, but not receipt. Following naltrexone, ACC activation increased during reward anticipation, but the finding did not reach statistical significance (Δ%BOLD 0.059 ± 0.030, *p* = 0.086).

### Individual-level reward modulation by opioid antagonism

**Figure 3:**
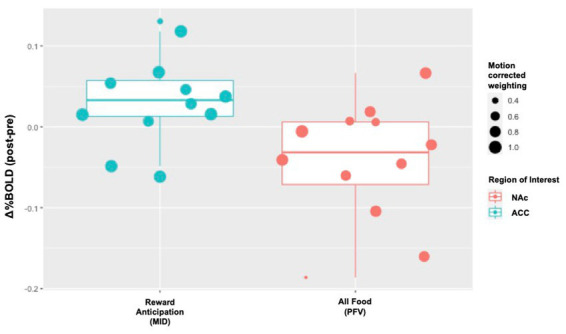
Individual-level Δ%BOLD following opioid antagonism. The boxplots display individual-level data points that represent the raw values of Δ%BOLD. The size of the data point corresponds with the amount of uncensored data for that individual (e., 1.0 means 100% of the data for that individual were uncensored; 0.8 means 80% of that individual’s data were uncensored and 20% censored due to motion). Following naltrexone, increased activity was seen in the anterior cingulate cortex, ACC, during reward anticipation (Cohen’s *d* 1.25, *p* = 0.086). Following naltrexone, reduced activity was seen in the nucleus accumbens, NAc, during passive food view task (Cohen’s *d* − 1.06, *p* = 0.048).

Individual level change in %BOLD for the two reward tasks is displayed in Figure 3. During reward anticipation (MID) and food-viewing (PFV), we anticipated increased activation in the ACC and reduced activation in the NAc, vmPFC and dlPFC following naltrexone. The proportion of individuals that did not show the hypothesized activation change post-naltrexone ranged from 17 to 33% across these four ROIs.

### Resting state functional connectivity

We explored the impact of naltrexone on rsFC in adolescents with eating disorders. Following naltrexone, group-level connectivity between reward and cortical regions was altered although variability was present within the cohort (NAc–L dlPFC: ΔFisher Z -0.089 ± 0.026, Cohen’s *d* − 1.29, *p* = 0.008; NAc – ACC: ΔFisher 0.039 ± 0.039, Cohen’s *d* − 0.82, *p* = 0.349) At the individual level, rsFC between the NAc and L dlPFC decreased in 75% of participants. Connectivity between the NAc and ACC decreased in 67% of participants.

## Discussion

Findings from this pilot study suggest that neuroimaging can detect acute opioid antagonism in youth with binge/purge eating disorders. This supports the notion that BOLD detection by fMRI is a promising pharmacodynamic biomarker for opioid antagonism in adolescents. Our study results suggest that naltrexone modulated reward processing through activation changes in the NAc, ACC, vmPFC and dlPFC. These ROIs contribute to the complexity of reward-related behavior, including anticipating reward (e.g., craving or urge), contemplating alternatives, and assigning reward value. For teens with binge/purge eating disorders, impulsive reward seeking (e.g., binge and purge behaviors) is associated with alterations in reward processing. Thus, control over the target behaviors of binge eating and purging may require tipping the balance toward improved inhibitory control and reduced urge. This is consistent with the acute activation changes seen post-naltrexone in this study.

Our targeted ROI analysis estimated large effects (Cohen’s *d* ≥ 0.8) of naltrexone that differed as a function of the reward probe (49). We expected to see substantial effects of naltrexone during the food-specific reward probe. Although we did see reduction in NAc activation when viewing pictures of food, we also saw reductions in NAc activation when viewing pictures of animals, suggesting that both food and non-food images were less rewarding post-naltrexone in our adolescent sample. The food specific reward probe did not outperform the probe eliciting more general reward (i.e., MID task). During the MID task, increased ACC activation was seen following naltrexone implying increased inhibitory or attentional control over future decisions and ability to consider reward alternatives compared to baseline (50–53). ACC alterations are seen in patients with eating disorders in response to food cues and seem to differ based on eating disorder type. Specifically, ACC activation (associated with inhibitory control) is detected in restrictive and recovered eating disorders compared with binge eating and/or purging eating disorders (54–56). Naltrexone–but not placebo–increased ACC activation in adults with substance use and obesity (20, 57). These findings support our overall hypothesis that opioid antagonism may increase inhibitory control of reward processing in adolescents with eating disorders, which may be important for the treatment of target behaviors.

Next, we used an ROI mask approach to case a wider, yet still targeted net. The chosen masks represent ROIs known to be rich in MOR, activated by exogenous opioids and previously associated with the reward tasks used (58, 59). Given our small sample size, it is not surprising that findings did not survive the strict correction for multiple comparisons. Rather, this analysis demonstrated that we did not miss any potentially significant findings with our targeted ROI-based approach in this pilot sample. Significant findings, however, may be detected in a larger sample size.

Beyond group-level performance, understanding individual-level data is necessary to describe the sensitivity of the pharmacodynamic biomarker. In our study, individual “non-responders,” defined as those who lack fMRI detection of acute reward modulation by opioid antagonism, comprised 17–33% of the cohort across the ROIs. This proportion is consistent with a prior proof-of-concept study using fMRI BOLD, where 10–20% of participants lacked the hypothesized brain activation changes in response to ketamine (60, 61). It is important to note that the use of the term “non-responders” in this context does not extend to clinical non-response. The relationship between acute neuroimaging changes and clinical response requires future research.

We also explored naltrexone’s ability to modulate rsFC in our sample of teens with eating disorders. Interestingly, the interconnectedness of NAc and dlPFC decreased at rest following naltrexone. Since enhanced connectivity in reward circuitry has been associated with eating disorder severity (62, 63), reduced rsFC following naltrexone may represent acute decoupling of these structures that may help ameliorate binge/purge behaviors. Consistent with this hypothesis, a longitudinal study of cortico-limbic FC in adolescents showed reduced connectivity over time, which was associated with a more “mature,” less reward driven phenotype (64). Alternatively, decreased connectivity between inhibitory control and reward circuitry has been shown in pre-adolescents with binge eating disorder relative to healthy controls (65). Two notable differences in our study exist: (1) we evaluated within-individual changes rather than comparison with healthy controls and (2) our study focused on adolescence, a developmental period associated with increased activity of the NAc relative to cortical regions. To our knowledge, there is no prior evidence on the impact of naltrexone on rsFC in adolescents.

fMRI detected BOLD or “pharmacoBOLD,” as previously coined, has been investigated as a non-invasive, low-risk biomarker of central nervous system functional target engagement in adults. PharmacoBOLD has been employed previously to detect opioid agonist activity (58) and glutaminergic engagement (60, 61), as well as to support dose exploration (66) and mechanistic proof of concept in early phase drug trials (20). Functional connectivity as another application of BOLD fMRI has also been explored previously as measure of acute neurological response to escitalopram in youth with anxiety (67). Our findings extend the role of fMRI to acute detection of opioid antagonism to adolescents with binge/purge eating disorders.

Findings from our pilot study should be interpreted within the following context. Our sample size is small yet was appropriate to meet the objectives of this pilot study (e.g., determine feasibility, estimate preliminary effect sizes) and enrolled predominately female patients with active binge/purge symptomatology (e.g., severity at or greater than nearly 3/4ths of youth with eating disorders) (26). We did not enroll healthy controls, as this was outside the scope of this pilot study. Future studies should evaluate differences in reward response to opioid antagonism between patients and healthy controls. The within-individual design was a strength that allowed us to detect acute change in %BOLD following a single dose of naltrexone in each participant, evaluating the magnitude of the effect as well as the percentage of those who did not demonstrate an effect. We reported mean ± standard error for Δ%BOLD along with the effect size and value of *p* in hypothesis-driven ROI. It is important to note that effect sizes detected in our pilot study are limited by the small sample and are meant to inform the design of future larger trials to validate the magnitude of the effect. As such, our findings are not intended to be interpreted through the lens of statistically significant value of ps; thus, value of *p* correction for multiple comparisons was not performed. With the within-individual, same day design, we did not counter-balance reward task presentation to avoid introducing additional complexity into the small sample. Intentionally, the post-naltrexone scan always occurred as the second scan of the day, thus habituation to the reward probes could occur. Yet, if habituation, and by extension attenuation of response, occurred with our design, that would suggest our findings underrepresent reward modulation by naltrexone. This could be rectified with a counter-balanced and randomized placebo-control trial, which is beyond the scope of the current study.

Our findings suggest that fMRI may be able to detect acute opioid reward system modulation in adolescents with binge/purge eating disorders. If validated in a larger, controlled study, the findings may facilitate the use of neuroimaging as a PD biomarker in adolescents with eating disorders. Such a biomarker would enable quantitative pharmacology trials to identify optimal dose and potentially guide efforts in drug discovery and repurposing.

## Data availability statement

The data supporting the conclusions of this manuscript may be made available upon request pending appropriate regulatory approvals (e.g., IRB). Requests to access the datasets should be directed to slstancil@cmh.edu.

## Ethics statement

This study was reviewed and approved by the Children’s Mercy Institutional Review Board. Written informed consent to participate in this study was obtained from the participant. If the participant was a minor, written informed consent was obtained from the participant’s legal guardian and assent was obtained from the participant.

## Author contributions

SS, AB, and SA-R contributed to conception of the study. SS, AB, SA-R, LM, MV contributed to the design of the study. H-WY, MB, and SS performed the statistical analysis. SS wrote the first draft of the manuscript. All authors contributed to manuscript revision, read, and approved the submitted version.

## Funding

This work was supported by a CTSA grant from NCATS awarded to Frontiers Clinical and Translational Science Institute # KL2TR002367 (SS) and UL1TR002366 (SS–pilot grant recipient). The contents are solely the responsibility of the authors and do not necessarily represent the official views of the NIH or NCATS.

## Conflict of interest

The authors declare that the research was conducted in the absence of any commercial or financial relationships that could be construed as a potential conflict of interest.

## Publisher’s note

All claims expressed in this article are solely those of the authors and do not necessarily represent those of their affiliated organizations, or those of the publisher, the editors and the reviewers. Any product that may be evaluated in this article, or claim that may be made by its manufacturer, is not guaranteed or endorsed by the publisher.
