# Supplementary material for: Potential biomarker of brain response to opioid antagonism in adolescents with eating disorders: a pilot study
Source: Front Psychiatry. 2023 Jul 10;14:1161032. doi: 10.3389/fpsyt.2023.1161032 (PMC10363723; doi:10.3389/fpsyt.2023.1161032)
Supplement: Supplementary file 1 [file Data_Sheet_1.DOCX]

**SUPPLEMENTARY METHODS**

**Biomarker of Brain Response to Opioid Antagonism in Adolescents with Eating Disorders**

Stephani L Stancil PhD APRN, Hung-Wen Yeh, PhD, Morgan G. Brucks, Amanda Bruce, PhD, Michaela Voss, MD, Susan Abdel-Rahman PharmD, William Brooks, PhD, Laura E. Martin, PhD

Neurosynth.org search strategy for regions of interest:

Bilateral Nucleus Accumbens

Function: Reward

Searched for: monetary incentive

Downloaded: monetary_incentive_association-test_z_FDR_0.01.nii

Right Ventromedial Prefrontal Cortex

Function: Reward

Searched for: monetary incentive

Downloaded: monetary_incentive_association-test_z_FDR_0.01.nii

Bilateral Anterior Cingulate Cortex

Function: Inhibition

Searched for: monetary incentive

Downloaded: monetary_incentive_association-test_z_FDR_0.01.nii

Left Dorsolateral Prefrontal Cortex

Function: Inhibition

Searched for: food

Downloaded: food_association-test_z_FDR_0.01.nii

| **Supplementary Table 1. Regions of interest for each task by location** | | |
| --- | --- | --- |
| **Task** | **ROI** | **MNI coordinates*** |
| **MID** | **NAc** | (L) -13, 5, -11 (R) 13, 7, -11 |
|  | **vmPFC** | (R) 7, 45, -17 |
|  | **ACC** | (L) -5, 29, 15 (R) 4, 30, 15 |
|  | **dlPFC** | (L) -23, 45, 32 |
| **PFV** | **NAc** | (L) -13, 5, -11 (R) 13, 7, -11 |
|  | **vmPFC** | (R) 7, 45, -17 |
|  | **ACC** | (L) -5, 29, 15 (R) 4, 30, 15 |
|  | **dlPFC** | (L) -23, 45, 32 |

Abbreviations: ACC, anterior cingulate cortex; dlPFC, dorsolateral prefrontal cortex; L, left; MID, monetary incentive delay; MNI: Montreal Neurological Institute; NAc, nucleus accumbens PFV, passive food view; R, right; ROI, region of interest; vmPFC, ventromedial prefrontal cortex
